# Challenges and Approaches of the Global Governance of Public Health Under COVID-19

**DOI:** 10.3389/fpubh.2021.727214

**Published:** 2021-11-08

**Authors:** Hu Zhang

**Affiliations:** East China University of Political Science and Law, Shanghai, China

**Keywords:** COVID-19, IHR2005, PHEIC, global governance, international public health

## Abstract

Public health events, as the common concern faced by the international community, call for the joint response from all mankind. The outbreak of the COVID-19 has highlighted the problems confronting the global governance of international public health, such as limited functions of international organizations and difficulties in achieving objectives, poor collaboration between governance subjects and their limited performance, overlapping legal basis of governance and blurred core function, and lack of solutions to special problems. The corresponding approaches can be taken to improve the efficiency of the governance of global public health, including supporting the role of international organizations to achieve the objectives, enhancing coordination among international governance subjects to form synergy, promoting the compliance with IHR2005 to avoid conflict of law application and upholding the vision of a community with a shared future for mankind to jointly respond to the special problems.

## Introduction

In December 2019, COVID-19 emerged and has since spread around the globe, infecting millions and leading to thousands of deaths. COVID-19 is characterized with strong transmissibility, infectiousness in its incubation period and threat to people's health and even life (1). Given the growing number of patients and reports of the epidemic in many countries around the world, the Committee of the World Health Organization (WHO), in accordance with *International Health Regulations (2005)* (IHR2005), agreed that the outbreak meets the criteria for a Public Health Emergency of International Concern (PHEIC) on the evening of 30 January 2020. It is thus clear that PHEIC becomes a global concern because of the growing interdependence of countries across the world in the face of disasters.

As early as the second half of the 19th century, the impact of international public health events had become one of the world's concerns (2). After the Second World War, the UN's Economic and Social Council held an international health conference in New York in July 1946, where the *Constitution of the World Health Organization* was adopted, along with a plan to establish the WHO. WHO, the world's organization to handle international health issues has, since its establishment, played an important guiding and promoting role in the cooperation to tackle international health issues, the prevention and control of infectious diseases, the improvement of theoretical research and practice of biomedicine, the development of health undertakings within member countries, and the improvement of people's health. Health has an intrinsic value to the individual and to the society (3). The preamble to the WHO Constitution embodies this aspiration. The development of society, along with the frequent international economic, trade and personnel exchanges, has also accelerated the globalization of public health. Public health has emerged as one of the most important issues of our global age. Globalization propels pathogens, placing us at risk (4). For centuries, several beliefs and political ideologies have existed on disease and its transmission (5). For instance, the first recorded smallpox epidemic began in Egypt in 1350 BCE. It reached China in 49 CE, Europe after 700, the Americas in 1,520, and Australia in 1,789. The bubonic plague, or “Black Death,” originated in Asia, but it spread to Europe in the fourteenth century, where it killed a fourth to a third of the population. Europeans carried diseases to the Americas in the fifteenth and sixteenth centuries that destroyed up to 95 percent of the indigenous population (6). From the 1980s just to the mid-twenty-first century, 2013–15, there were more than 12,000 major outbreaks of novel diseases, 215 infectious diseases, 44 million cases in 219 countries (4). Never before have public health problems been featured so urgently and comprehensively in the political, economic, and social dynamics of domestic and world affairs (7).

At the beginning of the twenty-first century there is widespread recognition that national and international health are inseparable (8). The COVID-19, in particular, spread to many countries in less than a month. The full extent of its impact on global economy, governance structures and livelihood of persons is unprecedented and huge but not fully known (5). These international public health events have left human efforts to control them in more uncertainty and difficulties. Governance challenges for global health have long fascinated legal scholars and political scientists (9). With the recent COVID-19 outbreak, it is high time to re-examine the current landscape of international health cooperation, which is underpinned by various legal norms, processes and institutions (10). The outbreak of COVID-19 will again test the effectiveness of IHR specifically, and the moral and legal legitimacy of the WHO as a global health agency more broadly (10). It has placed the issue of international public health governance once again into the focus of the international community.

## Challenges of COVID-19 to the International Publice Health

COVID-19 has been regarded as the “black swan” event of 2020, causing massive upheaval to businesses and impacting economic ecosystems on an unprecedented scale (11). While the WHO has been praised for its quickness in handling some of the more technical aspects of fighting a global pandemic, countries are taking their own approaches to the virus. In the case of China, early lockdown and forced quarantine measures seem to have been effective but such measures are not as easily implemented elsewhere (12). In South Korea, the focus has been on tracing the virus's spread through free, massive testing and then treating those who test positive. Social distancing has been encouraged through school closures, teleworking, and bans on large gatherings but forced quarantine has not been implemented (13). Italy and Spain, who delayed their containment strategies, have both favored less restrictive lockdown methods, though restrictions have increased as the situations in both countries have become more dire (14). In Germany, it is mainly local and regional governments that are responsible for health issues. The federal government's role is in most cases limited to coordinating the measures undertaken by the regional governments and to recommend a specific course of action for the whole country (15). Traditional global health leaders, such as the United States and the United Kingdom, proved unprepared for, and inept in responding to, the coronavirus (16). In the United Kingdom, a strategy of containment, delay, research, and mitigation have produced mixed results. Schools in the U.K. stayed open longer than countries on the continent and initial restrictive measures were aimed more at the most vulnerable like the elderly and those with comorbidities. While delaying actions may have allowed the U.K. to stave off some of the social and economic costs of the virus, it does not appear to have greatly lessened the spread of COVID-19 (17). The approach favored by several African countries has been stronger border protection *via* flight restrictions, visa denials, and 2-week quarantines for foreigners entering the country. While the numbers of infected persons remain lower on the African continent, it's unclear if this can be attributed to the tightening of borders (18).

At present, the main task of WHO and the state's governments is still to deal with the COVID-19, trying every means to control the epidemic and mitigate its harm. We do need to think locally, and act locally, but we also need to think and act globally. And ideas of global caring, global compassion, strong international institutions are really important (4). COVID-19 has challenged the sufficiency of even these significant global efforts (19), and the subsequent huge impacts impose new challenges to the global governance of international public health.

### Limited Functions of International Organizations and Difficulties in Achieving Objectives

IHR2005 is the main legal basis for the international community to govern public health, and WHO is at the core of global public health governance. International normative documents lay down the basis and guarantee for international organizations to play their roles, and also provide the ways for international organizations to perform their functions. However, the ability of WHO to affect national health decisions that impinge widely on economic and social life is limited by a world order dominated by independent nations (20). Obligations stipulated in the IHR2005 are based on seeking a balance between the national and the international community's interest. Meanwhile, the world's central health agency, WHO, lacks the legal authority to ensure equitable, needs-based distribution of medical supplies and equipment and vaccines and therapies, during a pandemic, heightening the vulnerability of people in poorer countries (21). By contrast, regimes such as the World Trade Organization give primacy to intellectual property protection rather than affordable biotechnologies (22). The World Bank and International Monetary Fund ushered in an era of user fees for health services and structural adjustment that diminished national health budgets (22). And laissez-faire capitalism gives carte blanche for transnational corporations to move to low-tax, low-regulation states, thus depleting domestic resources for health and failing to regulate corporate marketing, products, workplace safety, and environmental impacts that harm the public's health and safety (22).

The United Nations General Assembly (UNGA) and its Human Rights Council and United Nations Environment Programme (UNEP) have exerted far-reaching influence on the human rights and environmental protection issues involved in the response to public health events; the Office International Des Epizooties (OIE) and International Plant Protection Convention (IPPC) also enjoy the right to regulate the protection of plants and animals and organisms related to public health; the International Labor Organization (ILO) and International Maritime Organization (IMO) have adopted a large number of normative documents on labor and maritime navigation management issues arising from public health events. Therefore, they certainly do not provide us with warrant for a functionalist account of how governance arrangements for globalization would emerge, since the causal mechanism for selection seems even weaker at the global level than with respect to competition among states (23). The absence of a strong restraint mechanism will result in a lack of communication and coordination between the countries where PHEIC breaks out. Also, the role of international organizations in the joint response to PHEIC will be handicapped.

### Poor Collaboration Between Governance Subjects and the Limited Performance

Globalization has intensified economic interdependence, global communication, and international migration, giving new urgency to addressing health issues globally and inaugurating a new era in global health governance to replace the former international health governance (24). The shifts in global health law have driven it from its twentieth-century home in the lawmaking authorities of the WHO Constitution and toward a wider, more diverse range of international actors, including other United Nations (UN) agencies, the WTO, international arbitral tribunals, the UN Security Council, and large enterprises in health-related sectors like food, medicine, and tobacco (19). Governance in the more global world of the twenty-first century has become distinctly multi-layered and trans-scalar (25). There are many subjects involved in international public health governance.

The COVID-19 outbreak exposes the collective vulnerability against the invisible enemy that penetrate national borders with ease (10). In the face of PHEIC, concerted efforts are needed from international organizations, countries, non-governmental social organizations, enterprises and individuals. In the fight against COVID-19, poor coordination between the international public health governance subjects remained a problem.

First, the problem of uneven global development and scarce medical materials is particularly obvious. People in lower income countries are most vulnerable to the ravages of climate change (26), and have health systems and a social and economic infrastructure less able to deal with novel and emerging infectious diseases (27). Poor governance, violence, and political instability is most often felt by people in lower income countries. By comparison, costly gene therapy and precision medicine are most available in wealthier countries (21). Current signs are worrying; critically needed medical supplies and equipment are going primarily to the United States and countries in Europe, which can pay more (28). Huge numbers of the world's people, overwhelmingly poor and marginalized, have not benefited from global health improvements (21). These immense global health disparities are echoed in gaping inequities within countries-sometimes narrowing, but often expanding (21). Global forces, however, make it exceedingly hard to achieve health with justice. There are vast differences in the resources available to governments around the world. Low- and middle-income countries often lack the resources needed to safeguard the public's health, especially if there are significant disease burdens and large or fast-growing populations. The possibility of COVID-19 unleashing a catastrophe on countries with weak health and social support systems is frightfully real.

Second, no country acting alone can ensure all of the conditions for health. Think about transnational forces such as greenhouse gas emissions, or global rules and norms in areas such as trade and investment (22), or transnational corporations that actively seek low-tax, low-regulation destinations-or the rapid spread of communicable diseases, like COVID-19. Geographically, the development imbalance can be observed at both the inter-state and intra-state levels. Whenever an epidemic breaks out, it is the affected country or region that responds first and needs the active response from other countries. The states that bear the disproportionate burden of disease have the least capacity to do anything about it, and the states that have the wherewithal are deeply resistant to expending the political capital and economic resources necessary to truly make a difference to improve health outside their borders (29). Despite IHR2005's demands of its member's improvement in their domestic health conditions, developing countries, economically and technologically backward, are still unable to improve their domestic public health systems. Large cities and rural areas within the same country are faced with different challenges when responding to a pandemic, due to, for example, the density of population and the state of the infrastructure (15). It is difficult for them to provide adequate medical materials within a short time to deal with PHEIC. Global health with justice-a world where all people, wherever they live and whoever they are, can equally benefit from health improvements-remains seemingly over the horizon (21).

Furthermore, most states are faced with differences in economic and medical development between different regions, which results in varying ability to respond to PHEIC in different parts of that state. For the states short of a strong central government in the middle of the regulation, it is easy for the different areas of that state to respond to a crisis in an in-coordinate manner. Trade also impacts an individual nation's willingness to regulate public health and safety standards (30). These incentives generate friction for mechanisms of cooperation, including international law, that emphasize information sharing, science-based decision-making, and equitable access to health resources (16). Thus, the lack of cooperation or poor collaboration between the various governance subjects mentioned above seriously impaired the governance effect.

### Overlapping Legal Basis of Governance and Blurred Core Function

Handling of PHEIC may, due to some reasons, involve the application of overlapping international laws to the same event, and blur the main legal basis offered by IHR2005 and other treaties for the international community governance of public health. These treaties do not provide for any legal consequences or responsibility for non-compliance with those obligations (31). Problems of “institutional overload” and inconsistent standard setting are already emerging in international health (32), involving public health issues in trade concerns faces many barriers, including institutional resistance and a lack of coordination and resources (30). To balance health, trade and movement of people, IHR2005 empowers its members to enact laws to implement health policies in accordance with their own circumstances, on condition that they stick to the purposes of IHR2005 in accordance with article 3.4 of IHR2005.

The outbreak of COVID-19 influenced the globalization. When the progress of globalization comes to a halt, governments are finding their commitment to free trade no longer the first priority (33). Therefore, by calibrating health and trade interests, the IHR resonate with international trade law under the WTO, which also recognizes the state's right to restrict trade for health purposes but limits this right to ensure that restrictions are necessary (34). Articles 7 and 8 of the TRIPS Agreement could represent both “context” and “purpose” in interpreting other TRIPS provisions pursuant to article 31.1 of the Vienna Convention on the Law of Treaties and thereby temper the strong rights granted to intellectual property holders by the TRIPS Agreement. However, they are fairly vague and aspirational provisions and therefore would be unlikely to resolve difficulties where the drafting is unambiguous and the “ordinary meaning” of given treaty terms are clear, as is arguably the case for compulsory licensing in connection with patented pharmaceuticals (35). Although it has become the consensus of international organizations and regions to strengthen international cooperation in addressing global health issues, unified coordination is still insufficient on how to regulate and guarantee international cooperation. With a plethora of international organizations sharing lawmaking authority for global health and with other health actors engaged in the international legislative process, international lawmaking shows potential for fragmented, uncoordinated and inefficient sprawl (9). The most important structural shortcoming of IHR is the lack of enforceable sanctions. For example, if a country fails to explain why it has adopted more restrictive traffic and trade measures than those recommended by WHO, no legal consequences follow. Based on the experiences of handling COVID-19 up to the time of writing this paper, it is apparent that there are many operational problems with the IHR2005. The role of the IHR2005 seems not to be critical in guiding States Parties for tackling the outbreak (36). The manner in which the pandemic exposed controversies and gaps in international law suggests that global health governance lack an effective system of law (16).

### Lack of Solutions to Special Problems

The COVID-19 has many special problems which have been hitherto unknown because of its sudden and comprehensive nature. Concerns over potential erosions in democracy and respect for human rights caused by government responses to the COVID- 19 pandemic are situated among broader worries over worldwide democratic backsliding in recent years (37). As governments worldwide administer lockdowns, travel limitations, and other restrictions to respond to the COVID-19, some experts have warned of a “parallel epidemic” of government repression (38). Meanwhile, even when restrictions may be justified on the basis of public health, the manner of application and enforcement of these measures may raise human rights concerns in some cases (37). The increasing “securitization” of health law means it may become a primary instrument of abusive and arbitrary state power (39). For example, several states have deployed surreptitious cell phone technologies to track persons potentially infected with COVID-19 and their contacts (40). Take the Infodemic as an example. “Infodemic,” like epidemics (41), involves the rapid spread of information of all kinds, including rumors, gossip and unreliable information. The world is in an era of network connectivity, information flooding and rapid dissemination. Once a public health emergency breaks out in a certain place, it will quickly become the focus of the world, and there will be a variety of online comments mixed with rational analysis and impetuous noise. Infodemic is an important part of outbreak response. It encompasses three main areas: (1) monitoring and identifying health threats, (2) outbreaks investigation, and (3) actions for mitigation and control (41). Behind this there are two reasons. First, to prevent the spread of the epidemic, the affected cities and countries may adopt lockdown policy, which increases the expectation of facts and security concerns. Second, people will feel worried about reality, which will be of little help. After the outbreak of COVID-19, the public media and Internet have been full of different voices, seriously denting the morale and enthusiasm of the people affected by the epidemic to fight against the epidemic. In view of this kind of phenomenon, some states have promulgated the bounded restrictions on the exercise some human rights, which arouses disputes. Experts worry that some restrictions fail to meet necessary principles to ensure respect for human rights (42). Others contend that during the pandemic, there has been increased pressure on civil liberties, such as threats to freedom of opinion, discussion, press freedom for journalists covering the news and scientists who had different opinions on the results of their research or studies (43). States of emergency are built on the somewhat artificial dichotomy of norm and exception, which endorses a bifurcated approach to balancing the interests of societal goals and individual rights (43). Therefore, the extent to which COVID- 19-related restrictions represent a departure from past governance patterns also may vary between states (37).

## Improve the Global Governance of International Public Health

As far as the competencies of the respective local governments worldwide are concerned, the situation differs from state to state. In a highly centralized state, local entities will be less able and therefore less inclined to invest in international cooperation when dealing with a health crisis, than local or regional governments in a more decentralized, federal structure (15). Much will depend on the allocation of competencies in the individual State as far as health issues are concerned (15). These different policy responses will often be transformed into different legal actions, so these patterns are helpful in understanding the role of law in connection with the globalization of public health (44). Health justice, then, is a fundamentally global concept and requires health equality within and across countries and regions (45). To achieve health justice and to ensure the smooth and efficient response to similar public health events in the future, the aforementioned challenges facing the international community in dealing with the epidemic can be addressed from the perspective of global governance in the following aspects.

### Support the Role of International Organizations to Achieve the Objectives

The responsibility for implementing IHR2005 is shared by the states parties and WHO. As the largest international health organization, and one of the larger specialized agencies of the United Nations, the WHO has far-reaching responsibilities to address global public health based on the responsibilities assigned by its constitution and its affiliation with the United Nations (9). Article 2 of the Constitution lists 22 functions of the Organization which, given the size and scale of the COVID-19 pandemic, almost all seem relevant to the impact of the disease (5). The WHO has an unparalleled law-making power among the international organizations with lawmaking authority (10). With its amiable power and authority, the agency has an unequivocal power to influence international health policies, however, with the agency's visible reluctant to utilize its law-making power (46), commentators have observed that the WHO is more contented to act as a technical agency than embracing a leadership role in global health (46).

A framework convention on global health or a similar mechanism would not be easy to achieve, and it certainly would not provide an ideal solution, but at least a framework convention would go toward the heart of the problem-that is, it would address state's obligations to act outside their borders and thus establish the levels of commitment and the kinds of interventions necessary to make a meaningful difference for the world's population (29). In day-to-day public health governance, states parties should work actively with WHO, mobilize financial resources, and facilitate the implementation of their IHR2005 obligations; they must improve their national surveillance and response infrastructure so as to enable timely warning of public health risks and emergencies. In the event of a public health risk or emergency, the states parties shall promptly notify WHO of the relevant risks and circumstances; WHO should assess the situation in the country where the risk or event occurs, and establish a special event information website. During the duration of the risk or event, the states parties shall faithfully report to WHO on a daily basis for WHO to publish the data on the information website; WHO shall make relevant information available to all focal points of states parties and the public, along with progress, guidance and warnings. In the case of a particular outbreak, WHO should assign commissioners to the outbreak site for investigation, so as to make a better response. All the above functions shall be strictly observed and carried out by WHO and the states parties. In particular, the states parties shall provide all facilities and support to WHO for the organization to better perform its duties. Also, WHO is in the process of establishing specific indicators for core competency readiness, which it hopes will help to better gauge member state's preparedness to respond to a public health emergency (47).

At the local level, the handling of COVID-19 and control of its spread are supposed to be the duties of the governments in the respective jurisdictions. At the international level, WHO is supposed to work closely with governments and to lead the world to fight against the outbreak based on IHR2005 (36). No matter whether some of the criticism of the WHO's initial reaction to the crisis is justified or not, surely it is to the world's advantage to already have such a forum in order to share experiences and resources (15). In an interconnected world, driven by an increased rate of economic globalization, global governance of health is a contentious field, often fought with ideological disagreements (10). It is with this knowledge that at the special summit on COVID-9 the parties committed themselves to taking all necessary actions within their respective mandates with relevant international organizations, including WHO, expressing full support and commitment to further enhance WHO's role in coordinating international anti-epidemic actions (48).

### Enhance Coordination Among International Governance Subjects to Form Synergy

Global governance refers to formal and informal sets of arrangements in global politics insofar as it implies that states alone cannot manage global affairs, but have to acknowledge the contributions of international governmental organizations, non-governmental organizations, and multinational corporations (49). In addition, given the increasingly active role of the public and transnational enterprises in the governance of international affairs, transnational corporations and industry elites should also be included in the main subjects of global governance. Among these subjects, countries are the fundamental driving force to promote global governance of public health, and the role of WHO in the global governance of public health is to be a leader, the coordinator and the platform provider. Theoretically, the diversity of governance subjects is based on the principle of subordination. The influential role that non-state actors play within international society cannot be denied (49). It is self-evident that stopping the spread of and dealing with a pandemic that threatens the whole world necessitates international cooperation in order to be effective (15). International cooperation is critical to combatting pathogenic threats (16), due to the differences in economic development, medical supplies and scientific and technological personnel reserves between different countries and between different parts of a country, it requires effective collaboration between different governance subjects.

Set up virtual medical platform to remedy defects caused by gaps in development. In the event of PHEIC, some countries may be reluctant to export scarce raw materials and medical resources. Therefore, a strong international stockpile helps ensure the supply of medical resources and necessities, regardless of whether they are produced or stored in certain countries. There is a condition for global health with justice is an international order and transnational action that systematically advances the conditions for good health and for accountable governance, particularly for people in countries on the short end of global health disparities (21). It is advisable to set up a virtual warehouse under the framework of WHO. This virtual warehouse is not a real warehouse, so that there is no need for each member country to actually deposit its claimed materials. The virtual warehouse is mainly used to store scarce raw materials and medical resources. Health financing is a critical, but often neglected, component of global governance of health (10). The funding sources of the warehouse can be divided into the following categories. a. reserved assessed amount by states parties. Member states claim a corresponding amount of medical products in proportion to their contributions on a yearly basis; b. social donation. The virtual warehouse is open to the whole society, so that all organizations, enterprises and individuals can provide targeted donations; c. additional donation. Countries with national supply or domestic manufacturing capacity contribute additional medical supplies to the virtual warehouse in addition to their assessed amounts. WHO acts as the manager of the virtual warehouse. In the event of a major outbreak, the affected country or region may apply for allocation of resources in accordance with the procedures prescribed by WHO. WHO may, after reviewing and approving the application, according to the principle of proportion and cost, direct countries which are not affected by the outbreak to provide appropriate medical materials to affected are as so as to overcome the plight of insufficient medical facilities caused by regional development gaps.

Promote cooperation between countries. As a consequence of globalization, governments must turn increasingly to international cooperation to attain national public health objectives and achieve some control over the trans-boundary forces that affect their populations (32). As a result, countries in today's world have long formed an interdependent relationship. Interdependence leads to shared benefits, which in turn encourage mutual cooperation. Public health features universality, actuality and practicality in its application, because it involves the fundamental and pervasive aspects of people's lives, so it is easier for public health to be generally recognized and applied. Therefore, cooperation is conducive to the well-being of the people, and will achieve the cooperative positive effect. With the rapid outbreak of the novel coronavirus across the global, effective containment of the outbreak requires global concerted efforts (10). Leadership from heads of state and government could help propel these ideas onto national and global agendas, providing critical support (21). Having paid a disastrously heavy price, the international community has eventually woken up and strengthened joint efforts in combating the virus (50). The G20 Extraordinary Leader's Summit held on March 26 in response to the COVID-19 pandemic marked the formation of an international consensus, where leaders expressed their commitment to “task our top relevant officials to coordinate closely in support of the global efforts to counter the pandemic's impacts.” Countries should cooperate actively in the face of a public health event. First, the affected countries or regions should take measures as soon as possible to prevent the epidemic from spreading to other countries. Second, other countries should help the affected countries as much as possible. State and local health departments are key to protecting the nation from epidemics (51). In today's globalized world, to help other countries to break away from the impact of public health events is essentially to help oneself. To take China as an example, in its fighting against the sudden outbreak of COVID-19, China has received support and assistance from foreign governments, enterprises, non-governmental organizations as well as friendly people worldwide. As its domestic situation was gradually stabilizing, China began providing support and assistance in various forms to the WHO and related UN agencies, neighboring countries, developing nations, and even the United States and European countries, which has been widely praised by the international community (52).

Build an effective mechanism for wide participation of the whole society. While the WHO recognizes its responsibility as stated in the Constitution for nomenclature of diseases, it has also been mindful of its functions to co-ordinate with other UN Specialized Agencies and scientific and technical groups (5). The vast array of international health actors actively involved in global health cooperation, combined with people's widespread criticism of the United Nations and its specialized agencies, have led some commentators to suggest a diminishing role for intergovernmental organizations in global health governance. Achieving global health justice requires authentic cooperation because the production of health equity at the global and domestic levels involves interdependent parties. This task requires individuals and groups to embrace and successfully fulfill respective roles and responsibilities based on functions and needs and voluntary commitments (53). Some have emphasized a “power shift” from intergovernmental organizations to private-sector actors and the innovative health coalitions described above (54). Because public-private partnerships have proliferated since 2,000, the contractual relationships between firms, governments, and large health-oriented foundations will serve as a significant source of global health law (19). The dispersal of governance in contemporary history has occurred not only across different layers and scales of social relations from the local to the global, but also with the emergence of various regulatory mechanisms in private quarters alongside those in the public sector (25). People are demanding decent health services. They want caring, compassionate, and highly qualified professionals. They demand affordable access to essential medicines, vaccines, and medical devices (21). To effectively respond to major disease outbreak, in addition to the government and scientific research institutions, large pharmaceutical enterprises and research and development centers also play an active role with their advanced research resources, and strong capability of R&D on immune and anti-epidemic drug. What's more, in combating the epidemic, donations from all walks of life have greatly alleviated the plight of medical resource shortage and material shortage in the most-affected areas. Given today's high-tech development, technology companies that dominating artificial intelligence and medical drug development are an indispensable in the fight against infectious diseases. The large number of volunteers are also a solid force for countries to deal with the outbreak. Therefore, WHO and countries should actively support and encourage enterprises and individuals to make joint efforts.

### Promote Compliance With IHR2005 to Avoid Conflict of Law Application

International law is the foundation of the international order, without which there would be no order in the international community. The 2003 SARS outbreak killed 916 people in 32 countries and regions, according to figures released by WHO on August 15, 2003 (55). The challenge for post-Westphalian public health is to create the conditions necessary for the governance innovations practiced in the SARS outbreak to be refined, improved, expanded, and sustained to meet the ongoing threats pathogenic microbes present (56). WHO adopted a series of internal working regulations and the IHR2005, which requires all countries to develop and maintain core health system capacities (4). Designed to enhance international health cooperation and provide an international legal framework, the IHR2005 aims to prevent, protect against, control and provide a public health response to the international spread of disease in ways that are commensurate with and restricted to public health risks, and which avoid unnecessary interference with international traffic and trade. Global health law encompasses the legal norms, processes, and institutions needed to create the conditions for people throughout the world to attain the highest possible level of physical and mental health (57). The need for international law in global public health is greater, however, than the attitudes of any particular WHO administration. WHO needs to take international law more seriously because the structure of international politics places international law in a central position in state's attempts to deal with global problems (44). Although the IHR2005 is the main international agreement on infectious diseases, the regulations do not govern every challenge that disease events create (16). The role of international law in the global governance is to guarantee the international public interest.

The 2005 World Summit Outcome adopted by the UNGA solemnly declares. We acknowledge that good governance and the rule of law at the national and international levels are essential for sustained economic growth, sustainable development and the eradication of poverty and hunger. Therefore, it is significant to recognize the need for universal adherence to and implementation of the rule of law at both the national and international levels. Against the background of global governance, a feasible means of addressing problems of global concern and avoiding power struggles among nations is to set up an international code of conduct through international collaboration and, on this basis, establish a predicable international system and develop a just and effective model of global governance (58). IHR2005 is both the norms of international law to deal with international public health problems formed on this basis, and also the basis of the international order followed by various subjects of international public health governance. Obviously, public health is critical to almost all major global governance issues, including national and international security, trade and economic development, environmental protection and human rights. IHR2005 has changed the traditional ways international community handles public health issues, and provided an important platform for extensive international communication.

The frameworks and platforms created by IHR2005 not only support international cooperation for improved health, but also underpin the strengthening of health systems within members, so as to generate stronger horizontal and vertical health governance among and within members. This change has the potential to contribute significantly to the overall task of global governance, improvement of the health of its members and all mankind. In the field of trade in goods, some have argued that in response to such concerns countries should pursue legal actions through the WTO and Article XX (b) of the General Agreement on Tariffs and Trade in order to ban potentially dangerous imports until the safety of such goods can be effectively established (59). The interdependence between international and national law emphasized by global health jurisprudence tends to be more robust when law supports governance actions that integrate power and ideas in a coherent and sustainable manner (7). Documents adopted by other international organizations, although with institutions related to public health, contain no provision for PHEIC. Therefore, in the event of PHEIC, IHR2005 should be given more attention than other international normative documents, even if there is inconsistency in the application of specific international laws.

### Uphold the Vision of a Community With a Shared Future for Mankind to Jointly Respond to the Special Problems

PHEIC is the common “enemy” of all humankind, which calls for countries to stop distinguishing between one another, and uphold the vision of a community with a shared future for mankind to jointly respond to the emergency. No country can fight an international epidemic by itself and yet, with the rise of populism and nationalism, the idea of “my country first,” where there is such a focus on economics, trade and self-interest, and so little on common global security (4). Building a community with a shared future for mankind was put forward by China as a global governance plan for the world (60). Today, “Jointly promote the building of a community with a shared future for mankind” has appeared in some important bilateral political declarations. For example, *The Joint Statement of the People's Republic of China and the Russian Federation* on June 8, 2018, *The Qingdao declaration of the Council of heads of state of the SCO member states* on June 10, 2018, and *The Beijing Declaration on building a closer community of common destiny for China and Africa* on September 3, 2018. Moreover, the concept of “building a community with a shared future for mankind” has been written into UN resolutions and is being included into the fundamental principle of international law.

Regardless of whether a public emergency has been declared, the International Covenant on Civil and Political Rights (ICCPR) also allows for bounded restrictions on freedoms of movement, assembly, expression, and association when necessary to protect public health. Some human rights treaties allow for bounded restrictions on the exercise of some human rights to meet public health crises. Most notably, ICCPR provides for certain derogations and restrictions (37). Emergency powers generally allow government powers to promptly respond to public emergencies in order to restore order and national security by suspending the ordinary legal system (61). According to the International Center for Not-for-Profit Law, 46 countries have instituted laws, policies, or practices related to COVID-19 that affect free expression in some way (62). “State of emergency” is therefore a label that may provide instant legitimacy to the greater limitation of human rights by government (63). Many governments have instituted or carried out control on the media and free expression under the justification of preventing the spread of misinformation or disinformation about the virus, with both new and existing laws and policies providing government officials the authority to prohibit the spread of virus-related information deemed they deem to be false or harmful (37).

As to Infodemic mentioned before, the prevention and control of infectious diseases inevitably involve the reduction and restriction of rights, but sanitary measures that restrict individual rights must follow the principles of necessity and fairness. Public health authorities around the world have been legitimately concerned about disinformation during the COVID-19 pandemic. Unreliable information, particularly when disseminated by individuals with significant platforms, can cause grave harm, whether maliciously intended or not. WHO has stated that “successful management of infodemics will be based on (1) monitoring and identifying them, (2) analysis of them, and (3) control and mitigation measures” (41). In the twentieth century, the exercise of public health powers that infringe individual rights faded in developed countries as public health and healthcare systems improved (64).

Paragraph 1 of article 3 and article 32 under IHR2005 demonstrates the importance of human rights protection in international health governance. In order to explain the relation between good governance and human rights, Koch points out that the former is something to which the individual is entitled, whereas the latter is something that the authorities are under an obligation to uphold (49). Therefore, the IHR2005 not only stipulates the goals of international public health governance, but also incorporates the principle of human rights into its implementation system, thus constructing the public health system that integrates security, economy, people's livelihood, development and human dignity with the attributes of public goods, rather than the previous IHR2005 only limited to removing restrictions on trade and travel. Public opinions are the sum of the beliefs, attitudes, and emotions of the public in response to the phenomena and problems in the society, mixed with rational and irrational factors. However, how public opinions are developed determines whether their effects on social development and the course of events are positive or negative. In the modern society featuring highly developed Internet access, people's ideas and values are more diversified, so are their ways of receiving information and channels of spreading information. Also, compared with the traditional model, the development of public opinions is more rapid, and the truth and rumor also exert a more obvious double-edged sword effect.

Against this background, the importance of public opinions is self-evident. Therefore, WHO, as the provider of information and guidance for the global response to PHEIC, should, while ensuring timely release of accurate information, clarify the false information spread in the society, so that all people can feel optimistic about the situation through the spread of true information. Information management may be seen through the lens of government obligations and company responsibilities, particularly companies involved in Internet searching or social media. On Feb. 14, WHO officials met with representatives from more than a dozen U.S. technology companies, including Facebook, Google, Amazon and the major topic of discussion was how the companies are working to tamp down the spread of misinformation (65). In addition, all news media and other media should work hard to contain the spread of wrong information about the epidemic so as to avoid public panic which may affect the efforts for the prevention and control of the epidemic.

## Conclusion

International public health has long ago been a common problem troubling the international community, which call for the joint response from all mankind. COVID-19 and its subsequent huge impact impose new challenges to the global governance of international public health. Facing the severe public health crisis the world has suffered, and the prevention mechanism established by the IHR2005 has not achieved the expected results. Particularly the recommendations issued by the WHO have not been universally adopted by member states, which expose some urgent problems in the international public health governance system. COVID-19 and other PHEIC are the common “enemy” of all humankind, which requires states to have a strong sense of unity, and uphold the vision of a community with a shared future for mankind to jointly respond to the epidemic.

## Author Contributions

The author confirms being the sole contributor of this work and has approved it for publication.

## Funding

This work was supported by 2019 major project of National Social Science Foundation of China Research on the construction of legal system of socialist foreign relations with Chinese characteristics (19ZDA167).

## Conflict of Interest

The author declares that the research was conducted in the absence of any commercial or financial relationships that could be construed as a potential conflict of interest.

## Publisher's Note

All claims expressed in this article are solely those of the authors and do not necessarily represent those of their affiliated organizations, or those of the publisher, the editors and the reviewers. Any product that may be evaluated in this article, or claim that may be made by its manufacturer, is not guaranteed or endorsed by the publisher.
